# Bis[*N*,*N*-bis­(diphenyl­phosphan­yl)pentyl­amine-κ^2^ *P*,*P*′]platinum(II) bis­(hexa­fluoridophosphate) dichloro­methane disolvate

**DOI:** 10.1107/S1600536810025560

**Published:** 2010-07-14

**Authors:** Ilana Engelbrecht, Hendrik G. Visser, Andreas Roodt

**Affiliations:** a Department of Chemistry, University of the Free State, PO Box 339, Bloemfontein 9300, South Africa

## Abstract

The Pt^II^ atom in the title compound, [Pt(C_29_H_31_NP_2_)_2_](PF_6_)_2_·2CH_2_Cl_2_, is coordinated by four P atoms from two bis­(di­phenyl­phosphan­yl)pentyl­amine ligands with an average Pt—P distance of 2.300 (1) Å. The coordination around the Pt^II^ atom shows a highly distorted square-planar geometry, as evidenced by the P—Pt—P bite angles of 70.45 (3) and 70.64 (3)°. The asymmetric unit contains two hexa­fluoridophosphate ions, the metal complex and two dichloro­methane solvent mol­ecules. One of the chloride atoms of one of the dichloro­methane mol­ecules is disordered over two sites in a 0.515 (3):0.485 (3) ratio. C—H⋯F hydrogen bonds stabilize the crystal packing.

## Related literature

For related platinum(II) complexes, see: Farrar & Browning (1995 ▶); Dyson *et al.* (2004 ▶); Cloete *et al.* (2010 ▶). For related diphenyl­phosphanyl ligands, see: Keat *et al.* (1981 ▶); Cloete *et al.* (2008 ▶, 2009 ▶); Cotton *et al.* (1996 ▶); Fei *et al.* (2003 ▶). For applications of diphenyl­phosphanyl ligands and their metal complexes in homogeneous catalysis, see: Steyn *et al.* (1992 ▶, 1997 ▶); Otto *et al.* (1998 ▶); Roodt & Steyn (2000 ▶); Brink *et al.* (2010 ▶); Viljoen *et al.* (2008 ▶, 2009*a* ▶,*b* ▶, 2010 ▶); Steyn *et al.* (2008 ▶).
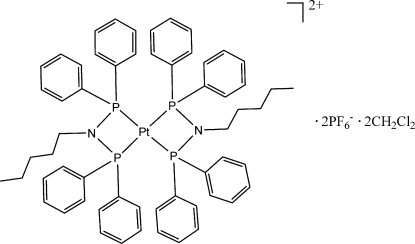


## Experimental

### 

#### Crystal data

[Pt(C_29_H_31_NP_2_)_2_](PF_6_)_2_·2CH_2_Cl_2_
*M* *_r_* = 1565.85
Monoclinic, 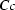

*a* = 11.3876 (10) Å
*b* = 24.283 (3) Å
*c* = 23.102 (2) Å
β = 97.669 (4)°
*V* = 6331.1 (11) Å^3^
*Z* = 4
Mo *K*α radiation
μ = 2.61 mm^−1^
*T* = 100 K
0.26 × 0.19 × 0.13 mm

#### Data collection

Bruker X8 APEXII 4K Kappa CCD diffractometer
Absorption correction: multi-scan (*SADABS*; Bruker, 2004 ▶) *T*_min_ = 0.550, *T*_max_ = 0.728
47664 measured reflections
14210 independent reflections
13180 reflections with *I* > 2σ(*I*)
*R*_int_ = 0.03

#### Refinement

*R*[*F* ^2^ > 2σ(*F* ^2^)] = 0.024
*wR*(*F* ^2^) = 0.047
*S* = 0.89
14210 reflections
767 parameters
2 restraints
H-atom parameters constrained
Δρ_max_ = 1.08 e Å^−3^
Δρ_min_ = −1.13 e Å^−3^
Absolute structure: Flack (1983 ▶), 6561 Friedel pairs
Flack parameter: 0.014 (2)

### 

Data collection: *APEX2* (Bruker, 2010 ▶); cell refinement: *SAINT-Plus* (Bruker, 2004 ▶); data reduction: *SAINT-Plus*; program(s) used to solve structure: *SHELXS97* (Sheldrick, 2008 ▶); program(s) used to refine structure: *SHELXL97* (Sheldrick, 2008 ▶); molecular graphics: *DIAMOND* (Brandenburg & Putz, 2005 ▶); software used to prepare material for publication: *WinGX* (Farrugia, 1999 ▶).

## Supplementary Material

Crystal structure: contains datablocks global, I. DOI: 10.1107/S1600536810025560/bt5278sup1.cif

Structure factors: contains datablocks I. DOI: 10.1107/S1600536810025560/bt5278Isup2.hkl

Additional supplementary materials:  crystallographic information; 3D view; checkCIF report

## Figures and Tables

**Table:** 

P1—Pt1	2.3063 (8)
P2—Pt1	2.2965 (8)
P3—Pt1	2.2994 (8)
P4—Pt1	2.2995 (8)

**Table:** 

P2—N1—P1	103.40 (13)
P4—N2—P3	103.15 (13)
N1—P1—Pt1	92.81 (9)
C41—P2—Pt1	118.82 (11)
C51—P3—Pt1	122.28 (11)
N2—P4—Pt1	93.11 (9)

**Table 2:** Hydrogen-bond geometry (Å, °)

*D*—H⋯*A*	*D*—H	H⋯*A*	*D*⋯*A*	*D*—H⋯*A*
C25—H25⋯F8	0.95	2.53	3.437 (4)	160
C55—H55⋯F2	0.95	2.49	3.079 (4)	120
C65—H65⋯F10	0.95	2.47	3.297 (4)	145
C83—H83⋯F11	0.95	2.37	3.267 (4)	158
C01—H01*B*⋯F2^i^	0.99	2.29	3.244 (5)	161
C01—H01*B*⋯F6^i^	0.99	2.4	3.150 (5)	132
C5—H5*C*⋯F11^ii^	0.98	2.51	3.196 (4)	127
C8—H8*A*⋯F3^iii^	0.99	2.54	3.450 (4)	153
C53—H53⋯F7^iv^	0.95	2.51	3.273 (4)	137
C63—H63⋯F4^v^	0.95	2.51	3.373 (4)	151
C73—H73⋯F9^vi^	0.95	2.53	3.426 (4)	156

Symmetry codes: (i) 
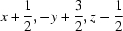
; (ii) 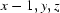; (iii) 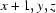; (iv) 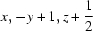; (v) 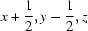; (vi) 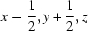.

**Fig. 1.**
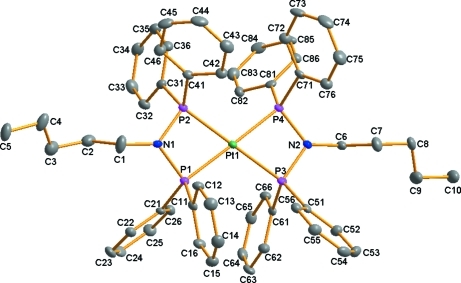
Molecular structure of the title compound. Displacement ellipsoids are drawn at the 50% probability level. Hydrogen atoms, solvent molecules and hexafluoridophosphate ions have been omitted for clarity and are displayed in Figure 2.

**Fig. 2.**
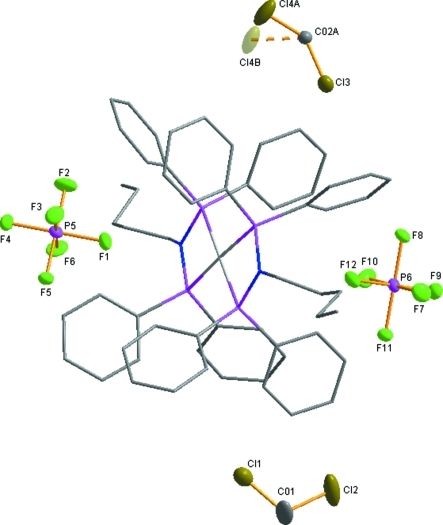
Numbering scheme of solvent molecules and hexafluoridophosphate ions. Displacement ellipsoids are drawn at the 50% probability level. Hydrogen atoms have been omitted for clarity.

## supplementary crystallographic information

### Comment

The synthesis of diphenylphosphanyl ligands and metal complexes thereof, forms part of ongoing research in the field of homogeneous catalysis (Steyn *et al.*, 1992, 1997; Otto *et al.*, 1998; Roodt & Steyn, 2000; Brink *et al.*, 2010; Viljoen *et al.*, 2008, 2009*a*,b, 2010; Steyn *et al.*, 2008). Colourless crystals of the title compound crystallize with two hexafluoridophosphate anions and two dichloromethane solvent molecules, of which one displays a 51% possitional disorder on one chloride atom. In the title compound, all bond distances and angles are considered to be normal and fall within the range reported for similar complexes (Farrar *et al.*, 1995; Dyson *et al.*, 2004; Cloete *et al.*, 2010). The square-planar geometry around the Pt^II^ metal centre is highly distorted with P1—Pt—P2 and P3—Pt—P4 bite angles of 70.45 (3) and 70.64 (3) °, respectively. The distorted tetrahedral angles of the P atoms, which range between 92.81 (9) and 122.28 (11) ° further illustrate the strain in the complex. The N atoms also deviate from the ideal tetrahedral configuration with P1—N1—P2 and P3—N2—P4 angles being 103.40 (13) and 103.15 (13) °, respectively. The distance between the N1 atom and the plane created by C1, P1 and P2 is -0.021 (2) ° whereas the distance of N2 and the plane created by C6, P3 and P4 shows a slightly bigger deviation of -0.122 (2) °. This shows that the N atom adopts an almost planar geometry with the two P atoms and the C atom attached to it in each case to accomodate the steric bulk of the phenyl groups and the alkyl group of the ligand. The intermolecular hydrogen bonds lead to a three-dimensional polymetric network obtained through C—H···F interactions.

### Experimental

[Pt(cod)Cl_2_] (20 mg, 0.0535 mmol) (cod = 1,5-cyclooctadiene) dissolved in the minimum amount of dichloromethane was added in a rapid dropwise manner to a solution of bis(diphenylphosphanyl)pentylamine (51.9 mg, 0.114 mmol) and NaPF_6_ (20 mg, 0.119 mmol) dissolved in the minimum volume of dichloromethane-methanol (1:1). After stirring for 20 min, the solvent was removed completely under reduced pressure. Dichloromethane was added until no further dissolution of solid was evident. The resulting heterogeneous mixture was filtered to remove the insoluble NaCl by-product. The colourless solid product was precipitated upon addition of methanol followed by a reduction in solvent volume under reduced pressure. The compound was isolated by filtration and washed with diethyl ether (10 cm^3^). Colourless crystals suitable for X-ray crystallography were obtained by the slow diffusion of diethyl ether into a solution of the product in dichloromethane at room temperature. (Crude yield: 39 mg, 66%) Spectroscopy data: ^1^H NMR (600 MHz, CD_2_Cl_2_): δ = 0.6 (t, 6H, CH_3_), 0.7 to 1.1 (m, 12H, 6 *x* CH_2_), 2.9 (t, 4H, N—CH_2_), 7.3 to 7.8 (m, 40H, Ar); ^31^P NMR (243 MHz, CD_2_Cl_2_): δ = 40.6 (t, ^1^J_Pt—P_ = 1063.0 Hz), -134.69 to -153.74 (m, PF_6_).

### Refinement

The methyl, methylene and aromatic H atoms were placed in geometrically idealized positions and constrained to ride on their parent atoms, with C—H = 0.95, 0.99 and 0.98Å and Uĩso~(H) = 1.5U~eq~(C) and 1.2U~eq~(C), respectively.The methyl groups were generated to fit the difference electron density and the groups were then refined as rigid rotors. The highest peak is located 0.06Å from C1 and the deepest hole is situated 0.06Å from C02B.

### Crystal data

**Table:**

[Pt(C_29_H_31_NP_2_)_2_](PF_6_)_2_·2CH_2_Cl_2_	*F*(000) = 3136
*M**_r_* = 1565.85	*D*_x_ = 1.643 Mg m^−^^3^
Monoclinic, *C**c*	Mo *K*α radiation, λ = 0.71073 Å
Hall symbol: C -2yc	Cell parameters from 9893 reflections
*a* = 11.3876 (10) Å	θ = 2.8–28.2°
*b* = 24.283 (3) Å	µ = 2.61 mm^−^^1^
*c* = 23.102 (2) Å	*T* = 100 K
β = 97.669 (4)°	Cuboid, colourless
*V* = 6331.1 (11) Å^3^	0.26 × 0.19 × 0.13 mm
*Z* = 4	

### Data collection

**Table:**

Bruker X8 APEXII 4K Kappa CCD diffractometer	14210 independent reflections
Radiation source: fine-focus sealed tube	13180 reflections with *I* > 2σ(*I*)
graphite	*R*_int_ = 0.03
ω and φ scans	θ_max_ = 28°, θ_min_ = 4.2°
Absorption correction: multi-scan (*SADABS*; Bruker, 2004)	*h* = −13→15
*T*_min_ = 0.550, *T*_max_ = 0.728	*k* = −32→32
47664 measured reflections	*l* = −30→30

### Refinement

**Table:**

Refinement on *F*^2^	Secondary atom site location: difference Fourier map
Least-squares matrix: full	Hydrogen site location: inferred from neighbouring sites
*R*[*F*^2^ > 2σ(*F*^2^)] = 0.024	H-atom parameters constrained
*wR*(*F*^2^) = 0.047	*w* = 1/[σ^2^(*F*_o_^2^) + (0.*P*)^2^] where *P* = (*F*_o_^2^ + 2*F*_c_^2^)/3
*S* = 0.89	(Δ/σ)_max_ = 0.045
14210 reflections	Δρ_max_ = 1.08 e Å^−^^3^
767 parameters	Δρ_min_ = −1.12 e Å^−^^3^
2 restraints	Absolute structure: Flack (1983), 6561 Friedel pairs
Primary atom site location: structure-invariant direct methods	Flack parameter: 0.014 (2)

### Special details

**Table:**

Experimental. The intensity data was collected on a Bruker X8 ApexII 4 K Kappa CCD diffractometer using an exposure time of 30 s/frame. A total of 1757 frames were collected with a frame width of 0.5° covering up to θ = 28.32° with 99.1% completeness accomplished.
Geometry. All s.u.'s (except the s.u. in the dihedral angle between two l.s. planes) are estimated using the full covariance matrix. The cell s.u.'s are taken into account individually in the estimation of s.u.'s in distances, angles and torsion angles; correlations between s.u.'s in cell parameters are only used when they are defined by crystal symmetry. An approximate (isotropic) treatment of cell s.u.'s is used for estimating s.u.'s involving l.s. planes.
Refinement. Refinement of *F*^2^ against ALL reflections. The weighted *R*-factor *wR* and goodness of fit *S* are based on *F*^2^, conventional *R*-factors *R* are based on *F*, with *F* set to zero for negative *F*^2^. The threshold expression of *F*^2^ > 2σ(*F*^2^) is used only for calculating *R*-factors(gt) *etc*. and is not relevant to the choice of reflections for refinement. *R*-factors based on *F*^2^ are statistically about twice as large as those based on *F*, and *R*- factors based on ALL data will be even larger.

### Fractional atomic coordinates and isotropic or equivalent isotropic displacement parameters (Å^2^)

**Table:**

	*x*	*y*	*z*	*U*_iso_*/*U*_eq_	Occ. (<1)
C1	0.1482 (3)	0.56240 (17)	0.77193 (14)	0.0287 (6)	
H1A	0.1064	0.5965	0.7575	0.034*	
H1B	0.0927	0.5402	0.7919	0.034*	
C01	0.8626 (5)	0.75498 (18)	0.64979 (17)	0.0559 (14)	
H01A	0.9186	0.7833	0.6393	0.067*	
H01B	0.79	0.7569	0.621	0.067*	
C2	0.1807 (3)	0.52966 (14)	0.71951 (14)	0.0255 (7)	
H2A	0.2232	0.5541	0.695	0.031*	
H2B	0.2349	0.4992	0.7338	0.031*	
C3	0.0724 (3)	0.50600 (16)	0.68260 (15)	0.0308 (8)	
H3A	0.0323	0.4804	0.707	0.037*	
H3B	0.0986	0.4843	0.6504	0.037*	
C4	−0.0175 (3)	0.54921 (16)	0.65640 (14)	0.0289 (9)	
H4A	0.0207	0.5737	0.6302	0.035*	
H4B	−0.0417	0.5721	0.6883	0.035*	
C5	−0.1280 (3)	0.52310 (16)	0.62195 (15)	0.0287 (6)	
H5A	−0.1046	0.5004	0.5904	0.043*	
H5B	−0.182	0.5522	0.6054	0.043*	
H5C	−0.1681	0.5001	0.6481	0.043*	
C6	0.7473 (3)	0.65347 (13)	1.03600 (13)	0.0153 (7)	
H6A	0.8068	0.6693	1.0132	0.018*	
H6B	0.7777	0.6174	1.0514	0.018*	
C7	0.7345 (3)	0.69112 (14)	1.08671 (14)	0.0229 (8)	
H7A	0.6786	0.6745	1.111	0.027*	
H7B	0.7008	0.7267	1.0717	0.027*	
C8	0.8540 (3)	0.70150 (14)	1.12475 (14)	0.0210 (7)	
H8A	0.9092	0.7181	1.1	0.025*	
H8B	0.8418	0.7286	1.1554	0.025*	
C9	0.9120 (3)	0.65016 (16)	1.15412 (16)	0.0335 (9)	
H9A	0.9334	0.6246	1.1238	0.04*	
H9B	0.8547	0.6312	1.1759	0.04*	
C10	1.0234 (3)	0.66455 (16)	1.19610 (15)	0.0301 (9)	
H10A	1.0794	0.6843	1.1748	0.045*	
H10B	1.0604	0.6306	1.2128	0.045*	
H10C	1.0018	0.6879	1.2276	0.045*	
C11	0.1754 (3)	0.54547 (12)	0.92209 (13)	0.0124 (6)	
C12	0.1015 (3)	0.59109 (13)	0.92072 (14)	0.0173 (7)	
H12	0.1063	0.6193	0.8926	0.021*	
C13	0.0210 (3)	0.59546 (14)	0.96012 (14)	0.0214 (7)	
H13	−0.0299	0.6266	0.9591	0.026*	
C14	0.0144 (3)	0.55456 (14)	1.00112 (14)	0.0223 (8)	
H14	−0.0404	0.5579	1.0285	0.027*	
C15	0.0874 (3)	0.50870 (14)	1.00257 (14)	0.0219 (7)	
H15	0.0821	0.4807	1.0308	0.026*	
C16	0.1678 (3)	0.50367 (14)	0.96310 (14)	0.0181 (8)	
H16	0.2174	0.4721	0.9638	0.022*	
C21	0.3353 (3)	0.47632 (12)	0.86595 (12)	0.0124 (6)	
C22	0.2482 (3)	0.43718 (13)	0.84764 (13)	0.0158 (7)	
H22	0.1667	0.4462	0.846	0.019*	
C23	0.2824 (3)	0.38498 (13)	0.83188 (14)	0.0201 (7)	
H23	0.2238	0.3581	0.8194	0.024*	
C24	0.4007 (3)	0.37181 (13)	0.83429 (14)	0.0202 (7)	
H24	0.423	0.3359	0.8235	0.024*	
C25	0.4881 (3)	0.41069 (13)	0.85241 (14)	0.0198 (7)	
H25	0.5695	0.4015	0.854	0.024*	
C26	0.4549 (3)	0.46288 (13)	0.86811 (12)	0.0159 (7)	
H26	0.5138	0.4896	0.8804	0.019*	
C31	0.4393 (3)	0.62230 (14)	0.75582 (14)	0.0138 (7)	
C32	0.4829 (3)	0.57081 (13)	0.74347 (13)	0.0179 (7)	
H32	0.4564	0.5389	0.7617	0.021*	
C33	0.5654 (3)	0.56589 (15)	0.70451 (14)	0.0237 (8)	
H33	0.5944	0.5306	0.6957	0.028*	
C34	0.6049 (3)	0.61239 (15)	0.67874 (16)	0.0231 (9)	
H34	0.661	0.6089	0.652	0.028*	
C35	0.5639 (3)	0.66405 (14)	0.69120 (13)	0.0199 (7)	
H35	0.5919	0.6957	0.6732	0.024*	
C36	0.4817 (3)	0.66947 (13)	0.73026 (13)	0.0162 (6)	
H36	0.4543	0.7049	0.7396	0.019*	
C41	0.2697 (3)	0.69369 (13)	0.80151 (13)	0.0139 (6)	
C42	0.2631 (3)	0.72902 (13)	0.84867 (14)	0.0170 (7)	
H42	0.2964	0.7182	0.8869	0.02*	
C43	0.2080 (3)	0.77987 (14)	0.83976 (15)	0.0238 (8)	
H43	0.2029	0.8037	0.8719	0.029*	
C44	0.1608 (3)	0.79582 (14)	0.78425 (15)	0.0245 (8)	
H44	0.1262	0.8313	0.7779	0.029*	
C45	0.1638 (3)	0.76030 (14)	0.73776 (15)	0.0236 (8)	
H45	0.1278	0.7708	0.6999	0.028*	
C46	0.2187 (3)	0.70949 (14)	0.74582 (14)	0.0206 (7)	
H46	0.2216	0.6855	0.7135	0.025*	
C51	0.4639 (3)	0.59396 (14)	1.06229 (14)	0.0128 (7)	
C52	0.5255 (3)	0.58111 (13)	1.11684 (13)	0.0175 (7)	
H52	0.6068	0.571	1.1202	0.021*	
C53	0.4689 (3)	0.58301 (14)	1.16598 (13)	0.0210 (7)	
H53	0.5116	0.5756	1.2034	0.025*	
C54	0.3483 (3)	0.59587 (13)	1.16053 (15)	0.0215 (7)	
H54	0.3083	0.5958	1.1941	0.026*	
C55	0.2871 (3)	0.60865 (13)	1.10647 (15)	0.0201 (8)	
H55	0.2054	0.6178	1.103	0.024*	
C56	0.3451 (3)	0.60810 (12)	1.05696 (14)	0.0164 (7)	
H56	0.3034	0.6174	1.0198	0.02*	
C61	0.6169 (2)	0.52730 (12)	1.00093 (12)	0.0113 (6)	
C62	0.5670 (3)	0.48035 (13)	1.02358 (13)	0.0168 (7)	
H62	0.5011	0.4839	1.0446	0.02*	
C63	0.6140 (3)	0.42899 (13)	1.01521 (14)	0.0194 (7)	
H63	0.58	0.3972	1.0302	0.023*	
C64	0.7105 (3)	0.42374 (14)	0.98505 (14)	0.0206 (7)	
H64	0.7423	0.3883	0.9793	0.025*	
C65	0.7610 (3)	0.46978 (13)	0.96313 (13)	0.0189 (7)	
H65	0.8281	0.4659	0.9431	0.023*	
C66	0.7136 (3)	0.52137 (13)	0.97039 (13)	0.0159 (7)	
H66	0.7472	0.5528	0.9545	0.019*	
C71	0.5542 (3)	0.74473 (12)	0.93612 (13)	0.0132 (6)	
C72	0.5523 (3)	0.77807 (13)	0.88622 (14)	0.0176 (7)	
H72	0.5783	0.7636	0.8519	0.021*	
C73	0.5127 (3)	0.83181 (13)	0.88716 (15)	0.0217 (7)	
H73	0.5089	0.854	0.8531	0.026*	
C74	0.4788 (3)	0.85312 (14)	0.93750 (16)	0.0257 (8)	
H74	0.4559	0.8907	0.9388	0.031*	
C75	0.4779 (3)	0.81994 (14)	0.98661 (15)	0.0257 (8)	
H75	0.4526	0.8346	1.021	0.031*	
C76	0.5137 (3)	0.76581 (13)	0.98528 (14)	0.0197 (7)	
H76	0.5106	0.7429	1.0184	0.024*	
C81	0.7146 (3)	0.67248 (13)	0.88803 (13)	0.0142 (6)	
C82	0.7258 (3)	0.63027 (13)	0.84829 (13)	0.0167 (7)	
H82	0.6677	0.602	0.8429	0.02*	
C83	0.8203 (3)	0.62906 (14)	0.81665 (14)	0.0225 (7)	
H83	0.8276	0.6	0.7898	0.027*	
C84	0.9043 (3)	0.67045 (14)	0.82435 (14)	0.0200 (7)	
H84	0.9685	0.6701	0.802	0.024*	
C85	0.8958 (3)	0.71218 (13)	0.86402 (13)	0.0192 (7)	
H85	0.955	0.7399	0.8696	0.023*	
C86	0.8009 (3)	0.71373 (13)	0.89587 (13)	0.0149 (6)	
H86	0.7945	0.7427	0.9229	0.018*	
N1	0.2516 (2)	0.57749 (10)	0.81489 (10)	0.0128 (5)	
N2	0.6347 (2)	0.64435 (10)	0.99603 (10)	0.0132 (5)	
F1	0.1335 (2)	0.71148 (9)	0.96105 (10)	0.0424 (6)	
F2	0.13372 (19)	0.70746 (9)	1.05954 (9)	0.0404 (6)	
F3	−0.02480 (17)	0.74020 (9)	1.00181 (8)	0.0330 (5)	
F4	0.0917 (2)	0.79785 (8)	1.06069 (8)	0.0385 (5)	
F5	0.0903 (2)	0.80160 (8)	0.96277 (8)	0.0345 (5)	
F6	0.24994 (19)	0.76911 (11)	1.02022 (10)	0.0551 (7)	
F7	0.72443 (19)	0.43486 (9)	0.74705 (9)	0.0375 (5)	
F8	0.78582 (17)	0.40997 (8)	0.84068 (8)	0.0255 (4)	
F9	0.91953 (17)	0.42305 (8)	0.77746 (8)	0.0314 (5)	
F10	0.90878 (19)	0.48342 (9)	0.85057 (8)	0.0385 (6)	
F11	0.84607 (19)	0.50836 (8)	0.75712 (9)	0.0349 (5)	
F12	0.71315 (19)	0.49579 (9)	0.82069 (10)	0.0341 (6)	
P1	0.29586 (7)	0.54624 (3)	0.87958 (3)	0.01116 (16)	
P2	0.34891 (7)	0.62997 (4)	0.81388 (3)	0.01159 (16)	
P3	0.53802 (7)	0.59136 (4)	0.99775 (3)	0.01165 (16)	
P4	0.59236 (7)	0.67322 (3)	0.92964 (3)	0.01178 (16)	
P5	0.11328 (8)	0.75458 (4)	1.01089 (4)	0.02094 (19)	
P6	0.81623 (8)	0.45921 (4)	0.79889 (4)	0.02016 (19)	
Pt1	0.441743 (9)	0.610768 (4)	0.906107 (7)	0.01061 (3)	
Cl1	0.82677 (9)	0.76853 (4)	0.71916 (4)	0.0404 (2)	
Cl2	0.92773 (14)	0.68887 (6)	0.64686 (5)	0.0751 (5)	
Cl3	0.30911 (8)	0.36043 (4)	0.97952 (4)	0.0320 (2)	
Cl4A	0.2246 (2)	0.36509 (14)	1.09592 (9)	0.0580 (9)	0.515 (3)
C02A	0.3267 (3)	0.34492 (15)	1.05375 (14)	0.0255 (7)	0.515 (3)
H02A	0.3339	0.3044	1.0574	0.031*	0.515 (3)
H02B	0.4037	0.3606	1.0711	0.031*	0.515 (3)
Cl4B	0.2955 (3)	0.40266 (14)	1.09242 (10)	0.0612 (9)	0.485 (3)
C02B	0.3267 (3)	0.34492 (15)	1.05375 (14)	0.0308 (8)	0.485 (3)
H02C	0.2727	0.3146	1.0612	0.037*	0.485 (3)
H02D	0.4091	0.3328	1.0666	0.037*	0.485 (3)

### Atomic displacement parameters (Å^2^)

**Table:**

	*U*^11^	*U*^22^	*U*^33^	*U*^12^	*U*^13^	*U*^23^
C1	0.0226 (13)	0.0425 (18)	0.0194 (12)	0.0050 (12)	−0.0034 (10)	−0.0088 (12)
C01	0.083 (4)	0.041 (3)	0.039 (3)	0.039 (3)	−0.010 (2)	0.001 (2)
C2	0.0335 (18)	0.0216 (18)	0.0222 (17)	0.0036 (14)	0.0069 (14)	0.0032 (14)
C3	0.041 (2)	0.031 (2)	0.0215 (17)	0.0028 (16)	0.0065 (15)	−0.0015 (15)
C4	0.0239 (19)	0.047 (3)	0.0160 (17)	0.0091 (17)	0.0024 (14)	−0.0047 (17)
C5	0.0226 (13)	0.0425 (18)	0.0194 (12)	0.0050 (12)	−0.0034 (10)	−0.0088 (12)
C6	0.0166 (16)	0.0123 (17)	0.0160 (16)	−0.0026 (12)	−0.0009 (13)	0.0025 (13)
C7	0.0225 (18)	0.0202 (19)	0.0250 (18)	0.0007 (14)	−0.0004 (15)	−0.0077 (15)
C8	0.0233 (18)	0.0207 (19)	0.0187 (17)	−0.0071 (14)	0.0020 (14)	−0.0071 (14)
C9	0.036 (2)	0.032 (2)	0.029 (2)	−0.0150 (17)	−0.0076 (17)	0.0064 (17)
C10	0.030 (2)	0.030 (2)	0.027 (2)	−0.0086 (16)	−0.0071 (16)	0.0046 (16)
C11	0.0101 (15)	0.0113 (16)	0.0160 (15)	−0.0021 (12)	0.0021 (12)	−0.0013 (12)
C12	0.0203 (17)	0.0128 (17)	0.0194 (17)	−0.0017 (13)	0.0050 (13)	0.0029 (13)
C13	0.0177 (17)	0.0214 (19)	0.0263 (19)	0.0037 (14)	0.0071 (14)	−0.0001 (15)
C14	0.0195 (18)	0.026 (2)	0.0230 (18)	−0.0019 (14)	0.0086 (14)	−0.0017 (15)
C15	0.0249 (19)	0.0208 (19)	0.0206 (17)	−0.0027 (14)	0.0051 (14)	0.0042 (14)
C16	0.0216 (18)	0.0113 (18)	0.0225 (19)	0.0015 (13)	0.0065 (15)	0.0006 (14)
C21	0.0153 (15)	0.0094 (16)	0.0124 (15)	−0.0020 (12)	0.0015 (12)	0.0025 (12)
C22	0.0170 (16)	0.0139 (17)	0.0170 (16)	−0.0016 (13)	0.0046 (13)	0.0000 (13)
C23	0.0229 (18)	0.0137 (17)	0.0240 (17)	−0.0065 (13)	0.0044 (14)	−0.0036 (14)
C24	0.0291 (19)	0.0087 (16)	0.0236 (18)	0.0034 (13)	0.0063 (14)	−0.0029 (13)
C25	0.0186 (17)	0.0160 (18)	0.0251 (18)	0.0054 (13)	0.0041 (14)	0.0001 (14)
C26	0.0176 (16)	0.0142 (17)	0.0163 (16)	−0.0019 (12)	0.0035 (12)	−0.0013 (13)
C31	0.0136 (16)	0.0159 (18)	0.0120 (16)	−0.0004 (13)	0.0020 (12)	0.0019 (14)
C32	0.0178 (17)	0.0132 (18)	0.0225 (17)	−0.0013 (13)	0.0026 (13)	0.0010 (14)
C33	0.0227 (18)	0.021 (2)	0.0282 (19)	0.0029 (14)	0.0056 (15)	−0.0078 (15)
C34	0.022 (2)	0.032 (2)	0.0168 (19)	−0.0028 (15)	0.0097 (16)	−0.0019 (16)
C35	0.0210 (17)	0.0212 (19)	0.0178 (16)	−0.0026 (14)	0.0034 (13)	0.0030 (14)
C36	0.0185 (16)	0.0130 (17)	0.0171 (16)	0.0027 (12)	0.0026 (13)	0.0009 (13)
C41	0.0145 (16)	0.0100 (16)	0.0179 (16)	0.0015 (12)	0.0049 (12)	0.0002 (13)
C42	0.0159 (16)	0.0156 (17)	0.0192 (16)	0.0038 (13)	0.0021 (13)	0.0019 (13)
C43	0.0237 (19)	0.020 (2)	0.030 (2)	0.0013 (14)	0.0115 (15)	−0.0041 (16)
C44	0.0227 (18)	0.0142 (18)	0.039 (2)	0.0065 (14)	0.0132 (16)	0.0079 (16)
C45	0.0247 (19)	0.022 (2)	0.0245 (18)	0.0082 (14)	0.0063 (14)	0.0113 (15)
C46	0.0222 (18)	0.0205 (19)	0.0191 (17)	0.0032 (14)	0.0021 (14)	0.0008 (14)
C51	0.0165 (17)	0.0075 (16)	0.0142 (16)	−0.0015 (14)	0.0009 (13)	−0.0018 (14)
C52	0.0168 (16)	0.0193 (18)	0.0165 (16)	0.0005 (13)	0.0030 (13)	0.0021 (14)
C53	0.0293 (19)	0.0210 (19)	0.0129 (16)	−0.0001 (15)	0.0033 (14)	0.0010 (14)
C54	0.031 (2)	0.0150 (18)	0.0218 (18)	−0.0013 (14)	0.0158 (15)	−0.0012 (14)
C55	0.0204 (19)	0.019 (2)	0.022 (2)	0.0018 (14)	0.0081 (16)	0.0014 (15)
C56	0.0178 (16)	0.0128 (17)	0.0184 (16)	−0.0006 (13)	0.0017 (13)	−0.0007 (13)
C61	0.0085 (14)	0.0116 (16)	0.0126 (15)	−0.0005 (11)	−0.0028 (11)	0.0004 (12)
C62	0.0182 (16)	0.0150 (17)	0.0174 (16)	0.0001 (13)	0.0030 (13)	0.0017 (13)
C63	0.0212 (17)	0.0093 (16)	0.0263 (18)	−0.0027 (13)	−0.0019 (14)	0.0015 (14)
C64	0.0247 (18)	0.0141 (18)	0.0215 (17)	0.0031 (14)	−0.0028 (14)	−0.0048 (14)
C65	0.0151 (16)	0.0218 (19)	0.0197 (17)	0.0032 (13)	0.0016 (13)	−0.0033 (14)
C66	0.0166 (16)	0.0131 (17)	0.0178 (16)	−0.0017 (12)	0.0013 (13)	0.0036 (13)
C71	0.0098 (15)	0.0085 (16)	0.0213 (17)	−0.0018 (11)	0.0025 (13)	−0.0012 (13)
C72	0.0159 (16)	0.0160 (18)	0.0214 (17)	0.0020 (13)	0.0043 (13)	0.0001 (14)
C73	0.0171 (17)	0.0118 (17)	0.036 (2)	0.0005 (13)	0.0035 (14)	0.0096 (15)
C74	0.0180 (17)	0.0115 (18)	0.047 (2)	0.0050 (13)	0.0043 (16)	−0.0001 (16)
C75	0.0254 (19)	0.020 (2)	0.032 (2)	0.0046 (14)	0.0049 (15)	−0.0057 (16)
C76	0.0208 (18)	0.0157 (18)	0.0228 (18)	0.0010 (13)	0.0033 (14)	0.0007 (14)
C81	0.0172 (16)	0.0126 (16)	0.0128 (15)	0.0037 (12)	0.0016 (12)	0.0057 (13)
C82	0.0206 (17)	0.0123 (16)	0.0163 (16)	−0.0002 (13)	0.0000 (13)	−0.0009 (13)
C83	0.0265 (19)	0.0193 (18)	0.0219 (18)	0.0061 (14)	0.0043 (15)	−0.0014 (14)
C84	0.0128 (15)	0.0237 (19)	0.0251 (18)	0.0083 (14)	0.0086 (13)	0.0070 (15)
C85	0.0169 (16)	0.0152 (18)	0.0254 (18)	−0.0008 (13)	0.0018 (13)	0.0081 (14)
C86	0.0161 (16)	0.0113 (17)	0.0172 (16)	0.0009 (12)	0.0012 (13)	0.0000 (13)
N1	0.0164 (14)	0.0105 (14)	0.0117 (13)	−0.0018 (10)	0.0029 (10)	0.0025 (11)
N2	0.0155 (14)	0.0094 (14)	0.0144 (13)	−0.0031 (10)	0.0008 (11)	0.0002 (11)
F1	0.0632 (16)	0.0221 (13)	0.0480 (14)	0.0013 (11)	0.0298 (12)	−0.0063 (11)
F2	0.0393 (14)	0.0381 (14)	0.0445 (14)	0.0116 (10)	0.0078 (11)	0.0248 (11)
F3	0.0222 (11)	0.0453 (14)	0.0309 (11)	−0.0060 (9)	0.0019 (9)	−0.0027 (10)
F4	0.0682 (16)	0.0261 (13)	0.0226 (11)	−0.0068 (11)	0.0115 (10)	−0.0082 (9)
F5	0.0593 (15)	0.0232 (12)	0.0223 (11)	−0.0004 (10)	0.0107 (10)	0.0041 (9)
F6	0.0236 (13)	0.089 (2)	0.0527 (15)	−0.0191 (12)	0.0053 (11)	0.0092 (14)
F7	0.0388 (13)	0.0365 (14)	0.0330 (12)	−0.0031 (10)	−0.0100 (10)	−0.0004 (10)
F8	0.0283 (11)	0.0167 (10)	0.0319 (11)	0.0016 (8)	0.0058 (9)	0.0050 (9)
F9	0.0334 (12)	0.0306 (12)	0.0323 (12)	0.0132 (9)	0.0122 (9)	0.0028 (9)
F10	0.0381 (13)	0.0467 (15)	0.0308 (12)	−0.0201 (11)	0.0048 (10)	−0.0113 (11)
F11	0.0504 (14)	0.0223 (12)	0.0369 (12)	0.0045 (10)	0.0239 (10)	0.0066 (10)
F12	0.0343 (13)	0.0212 (12)	0.0514 (15)	0.0085 (9)	0.0224 (11)	0.0054 (11)
P1	0.0111 (4)	0.0094 (4)	0.0131 (4)	−0.0002 (3)	0.0021 (3)	0.0003 (3)
P2	0.0131 (4)	0.0091 (4)	0.0128 (4)	−0.0005 (3)	0.0024 (3)	0.0003 (3)
P3	0.0139 (4)	0.0082 (4)	0.0131 (4)	0.0003 (3)	0.0030 (3)	0.0008 (3)
P4	0.0139 (4)	0.0084 (4)	0.0130 (4)	−0.0007 (3)	0.0016 (3)	0.0009 (3)
P5	0.0224 (5)	0.0167 (5)	0.0244 (5)	−0.0013 (3)	0.0057 (4)	0.0022 (4)
P6	0.0210 (5)	0.0155 (5)	0.0243 (5)	0.0015 (3)	0.0045 (4)	−0.0012 (4)
Pt1	0.01167 (5)	0.00794 (5)	0.01223 (5)	−0.00055 (6)	0.00167 (3)	0.00060 (6)
Cl1	0.0451 (6)	0.0342 (6)	0.0393 (5)	0.0035 (5)	−0.0036 (5)	0.0046 (5)
Cl2	0.1083 (12)	0.0499 (8)	0.0559 (8)	0.0418 (8)	−0.0303 (7)	−0.0227 (6)
Cl3	0.0380 (5)	0.0263 (5)	0.0310 (5)	0.0089 (4)	0.0017 (4)	0.0022 (4)
Cl4A	0.0499 (15)	0.098 (2)	0.0262 (11)	0.0341 (15)	0.0077 (10)	−0.0048 (12)
C02A	0.0335 (18)	0.0216 (18)	0.0222 (17)	0.0036 (14)	0.0069 (14)	0.0032 (14)
Cl4B	0.072 (2)	0.081 (2)	0.0297 (12)	0.0381 (17)	0.0039 (12)	−0.0141 (13)
C02B	0.041 (2)	0.031 (2)	0.0215 (17)	0.0028 (16)	0.0065 (15)	−0.0015 (15)

### Geometric parameters (Å, °)

**Table:**

C1—N1	1.481 (4)	C43—H43	0.95
C1—C2	1.535 (5)	C44—C45	1.381 (5)
C1—H1A	0.99	C44—H44	0.95
C1—H1B	0.99	C45—C46	1.385 (4)
C01—Cl1	1.737 (4)	C45—H45	0.95
C01—Cl2	1.774 (4)	C46—H46	0.95
C01—H01A	0.99	C51—C56	1.386 (4)
C01—H01B	0.99	C51—C52	1.394 (4)
C2—C3	1.516 (5)	C51—P3	1.810 (3)
C2—H2A	0.99	C52—C53	1.380 (4)
C2—H2B	0.99	C52—H52	0.95
C3—C4	1.533 (5)	C53—C54	1.397 (5)
C3—H3A	0.99	C53—H53	0.95
C3—H3B	0.99	C54—C55	1.382 (5)
C4—C5	1.534 (5)	C54—H54	0.95
C4—H4A	0.99	C55—C56	1.395 (5)
C4—H4B	0.99	C55—H55	0.95
C5—H5A	0.98	C56—H56	0.95
C5—H5B	0.98	C61—C66	1.393 (4)
C5—H5C	0.98	C61—C62	1.405 (4)
C6—N2	1.493 (4)	C61—P3	1.793 (3)
C6—C7	1.508 (4)	C62—C63	1.381 (4)
C6—H6A	0.99	C62—H62	0.95
C6—H6B	0.99	C63—C64	1.383 (5)
C7—C8	1.539 (4)	C63—H63	0.95
C7—H7A	0.99	C64—C65	1.384 (4)
C7—H7B	0.99	C64—H64	0.95
C8—C9	1.527 (5)	C65—C66	1.383 (4)
C8—H8A	0.99	C65—H65	0.95
C8—H8B	0.99	C66—H66	0.95
C9—C10	1.531 (5)	C71—C76	1.380 (4)
C9—H9A	0.99	C71—C72	1.406 (4)
C9—H9B	0.99	C71—P4	1.801 (3)
C10—H10A	0.98	C72—C73	1.382 (4)
C10—H10B	0.98	C72—H72	0.95
C10—H10C	0.98	C73—C74	1.375 (5)
C11—C12	1.389 (4)	C73—H73	0.95
C11—C16	1.399 (4)	C74—C75	1.393 (5)
C11—P1	1.791 (3)	C74—H74	0.95
C12—C13	1.379 (4)	C75—C76	1.378 (4)
C12—H12	0.95	C75—H75	0.95
C13—C14	1.381 (4)	C76—H76	0.95
C13—H13	0.95	C81—C82	1.393 (4)
C14—C15	1.387 (5)	C81—C86	1.397 (4)
C14—H14	0.95	C81—P4	1.794 (3)
C15—C16	1.381 (4)	C82—C83	1.379 (4)
C15—H15	0.95	C82—H82	0.95
C16—H16	0.95	C83—C84	1.382 (5)
C21—C26	1.395 (4)	C83—H83	0.95
C21—C22	1.397 (4)	C84—C85	1.378 (4)
C21—P1	1.795 (3)	C84—H84	0.95
C22—C23	1.389 (4)	C85—C86	1.387 (4)
C22—H22	0.95	C85—H85	0.95
C23—C24	1.378 (5)	C86—H86	0.95
C23—H23	0.95	N1—P2	1.691 (3)
C24—C25	1.394 (5)	N1—P1	1.692 (3)
C24—H24	0.95	N2—P4	1.697 (3)
C25—C26	1.385 (4)	N2—P3	1.697 (3)
C25—H25	0.95	F1—P5	1.595 (2)
C26—H26	0.95	F2—P5	1.599 (2)
C31—C32	1.389 (4)	F3—P5	1.597 (2)
C31—C36	1.403 (4)	F4—P5	1.601 (2)
C31—P2	1.806 (3)	F5—P5	1.591 (2)
C32—C33	1.390 (4)	F6—P5	1.582 (2)
C32—H32	0.95	F7—P6	1.595 (2)
C33—C34	1.380 (5)	F8—P6	1.603 (2)
C33—H33	0.95	F9—P6	1.598 (2)
C34—C35	1.382 (5)	F10—P6	1.596 (2)
C34—H34	0.95	F11—P6	1.599 (2)
C35—C36	1.391 (4)	F12—P6	1.606 (2)
C35—H35	0.95	P1—Pt1	2.3063 (8)
C36—H36	0.95	P2—Pt1	2.2965 (8)
C41—C46	1.393 (4)	P3—Pt1	2.2994 (8)
C41—C42	1.397 (4)	P4—Pt1	2.2995 (8)
C41—P2	1.795 (3)	Cl3—C02A	1.741 (3)
C42—C43	1.388 (4)	Cl4A—C02A	1.687 (4)
C42—H42	0.95	C02A—H02A	0.99
C43—C44	1.378 (5)	C02A—H02B	0.99
			
N1—C1—C2	113.8 (3)	C53—C52—C51	120.1 (3)
N1—C1—H1A	108.8	C53—C52—H52	119.9
C2—C1—H1A	108.8	C51—C52—H52	119.9
N1—C1—H1B	108.8	C52—C53—C54	119.7 (3)
C2—C1—H1B	108.8	C52—C53—H53	120.2
H1A—C1—H1B	107.7	C54—C53—H53	120.2
Cl1—C01—Cl2	111.0 (2)	C55—C54—C53	120.2 (3)
Cl1—C01—H01A	109.4	C55—C54—H54	119.9
Cl2—C01—H01A	109.4	C53—C54—H54	119.9
Cl1—C01—H01B	109.4	C54—C55—C56	120.1 (3)
Cl2—C01—H01B	109.4	C54—C55—H55	119.9
H01A—C01—H01B	108	C56—C55—H55	119.9
C3—C2—C1	112.1 (3)	C51—C56—C55	119.5 (3)
C3—C2—H2A	109.2	C51—C56—H56	120.2
C1—C2—H2A	109.2	C55—C56—H56	120.2
C3—C2—H2B	109.2	C66—C61—C62	119.4 (3)
C1—C2—H2B	109.2	C66—C61—P3	119.6 (2)
H2A—C2—H2B	107.9	C62—C61—P3	119.6 (2)
C2—C3—C4	114.4 (3)	C63—C62—C61	119.8 (3)
C2—C3—H3A	108.7	C63—C62—H62	120.1
C4—C3—H3A	108.7	C61—C62—H62	120.1
C2—C3—H3B	108.7	C62—C63—C64	120.2 (3)
C4—C3—H3B	108.7	C62—C63—H63	119.9
H3A—C3—H3B	107.6	C64—C63—H63	119.9
C3—C4—C5	112.4 (3)	C63—C64—C65	120.5 (3)
C3—C4—H4A	109.1	C63—C64—H64	119.8
C5—C4—H4A	109.1	C65—C64—H64	119.8
C3—C4—H4B	109.1	C66—C65—C64	120.0 (3)
C5—C4—H4B	109.1	C66—C65—H65	120
H4A—C4—H4B	107.9	C64—C65—H65	120
C4—C5—H5A	109.5	C65—C66—C61	120.2 (3)
C4—C5—H5B	109.5	C65—C66—H66	119.9
H5A—C5—H5B	109.5	C61—C66—H66	119.9
C4—C5—H5C	109.5	C76—C71—C72	119.4 (3)
H5A—C5—H5C	109.5	C76—C71—P4	122.5 (2)
H5B—C5—H5C	109.5	C72—C71—P4	117.7 (2)
N2—C6—C7	114.0 (3)	C73—C72—C71	120.0 (3)
N2—C6—H6A	108.8	C73—C72—H72	120
C7—C6—H6A	108.8	C71—C72—H72	120
N2—C6—H6B	108.8	C74—C73—C72	119.9 (3)
C7—C6—H6B	108.8	C74—C73—H73	120.1
H6A—C6—H6B	107.7	C72—C73—H73	120.1
C6—C7—C8	111.9 (3)	C73—C74—C75	120.3 (3)
C6—C7—H7A	109.2	C73—C74—H74	119.8
C8—C7—H7A	109.2	C75—C74—H74	119.8
C6—C7—H7B	109.2	C76—C75—C74	120.0 (3)
C8—C7—H7B	109.2	C76—C75—H75	120
H7A—C7—H7B	107.9	C74—C75—H75	120
C9—C8—C7	114.7 (3)	C75—C76—C71	120.3 (3)
C9—C8—H8A	108.6	C75—C76—H76	119.8
C7—C8—H8A	108.6	C71—C76—H76	119.8
C9—C8—H8B	108.6	C82—C81—C86	119.2 (3)
C7—C8—H8B	108.6	C82—C81—P4	120.4 (2)
H8A—C8—H8B	107.6	C86—C81—P4	120.4 (2)
C8—C9—C10	111.6 (3)	C83—C82—C81	120.8 (3)
C8—C9—H9A	109.3	C83—C82—H82	119.6
C10—C9—H9A	109.3	C81—C82—H82	119.6
C8—C9—H9B	109.3	C82—C83—C84	119.4 (3)
C10—C9—H9B	109.3	C82—C83—H83	120.3
H9A—C9—H9B	108	C84—C83—H83	120.3
C9—C10—H10A	109.5	C85—C84—C83	120.8 (3)
C9—C10—H10B	109.5	C85—C84—H84	119.6
H10A—C10—H10B	109.5	C83—C84—H84	119.6
C9—C10—H10C	109.5	C84—C85—C86	120.1 (3)
H10A—C10—H10C	109.5	C84—C85—H85	120
H10B—C10—H10C	109.5	C86—C85—H85	120
C12—C11—C16	120.1 (3)	C85—C86—C81	119.7 (3)
C12—C11—P1	119.0 (2)	C85—C86—H86	120.1
C16—C11—P1	120.0 (2)	C81—C86—H86	120.1
C13—C12—C11	120.0 (3)	C1—N1—P2	129.9 (2)
C13—C12—H12	120	C1—N1—P1	126.7 (2)
C11—C12—H12	120	P2—N1—P1	103.40 (13)
C12—C13—C14	119.9 (3)	C6—N2—P4	127.9 (2)
C12—C13—H13	120	C6—N2—P3	127.2 (2)
C14—C13—H13	120	P4—N2—P3	103.15 (13)
C13—C14—C15	120.4 (3)	N1—P1—C11	109.23 (13)
C13—C14—H14	119.8	N1—P1—C21	108.74 (13)
C15—C14—H14	119.8	C11—P1—C21	108.33 (14)
C16—C15—C14	120.2 (3)	N1—P1—Pt1	92.81 (9)
C16—C15—H15	119.9	C11—P1—Pt1	116.03 (10)
C14—C15—H15	119.9	C21—P1—Pt1	120.08 (10)
C15—C16—C11	119.3 (3)	N1—P2—C41	109.61 (14)
C15—C16—H16	120.3	N1—P2—C31	111.84 (15)
C11—C16—H16	120.3	C41—P2—C31	107.14 (15)
C26—C21—C22	120.1 (3)	N1—P2—Pt1	93.19 (9)
C26—C21—P1	118.8 (2)	C41—P2—Pt1	118.82 (11)
C22—C21—P1	120.8 (2)	C31—P2—Pt1	115.52 (11)
C23—C22—C21	119.2 (3)	N2—P3—C61	109.63 (14)
C23—C22—H22	120.4	N2—P3—C51	111.60 (14)
C21—C22—H22	120.4	C61—P3—C51	106.41 (15)
C24—C23—C22	120.5 (3)	N2—P3—Pt1	93.10 (9)
C24—C23—H23	119.7	C61—P3—Pt1	112.97 (10)
C22—C23—H23	119.7	C51—P3—Pt1	122.28 (11)
C23—C24—C25	120.6 (3)	N2—P4—C81	109.44 (13)
C23—C24—H24	119.7	N2—P4—C71	111.45 (13)
C25—C24—H24	119.7	C81—P4—C71	105.50 (14)
C26—C25—C24	119.3 (3)	N2—P4—Pt1	93.11 (9)
C26—C25—H25	120.3	C81—P4—Pt1	118.32 (11)
C24—C25—H25	120.3	C71—P4—Pt1	118.34 (10)
C25—C26—C21	120.3 (3)	F6—P5—F5	90.13 (13)
C25—C26—H26	119.9	F6—P5—F1	90.59 (14)
C21—C26—H26	119.9	F5—P5—F1	89.57 (12)
C32—C31—C36	119.7 (3)	F6—P5—F3	179.62 (16)
C32—C31—P2	120.0 (3)	F5—P5—F3	89.88 (12)
C36—C31—P2	119.4 (3)	F1—P5—F3	89.79 (12)
C31—C32—C33	120.1 (3)	F6—P5—F2	90.89 (13)
C31—C32—H32	120	F5—P5—F2	178.86 (13)
C33—C32—H32	120	F1—P5—F2	90.94 (12)
C34—C33—C32	119.8 (3)	F3—P5—F2	89.10 (12)
C34—C33—H33	120.1	F6—P5—F4	89.92 (14)
C32—C33—H33	120.1	F5—P5—F4	90.26 (11)
C33—C34—C35	121.0 (3)	F1—P5—F4	179.46 (15)
C33—C34—H34	119.5	F3—P5—F4	89.70 (12)
C35—C34—H34	119.5	F2—P5—F4	89.22 (12)
C34—C35—C36	119.8 (3)	F7—P6—F10	179.64 (15)
C34—C35—H35	120.1	F7—P6—F9	89.97 (12)
C36—C35—H35	120.1	F10—P6—F9	89.67 (12)
C35—C36—C31	119.7 (3)	F7—P6—F11	89.68 (12)
C35—C36—H36	120.2	F10—P6—F11	90.24 (12)
C31—C36—H36	120.2	F9—P6—F11	89.93 (11)
C46—C41—C42	119.5 (3)	F7—P6—F8	90.17 (12)
C46—C41—P2	121.6 (2)	F10—P6—F8	89.90 (11)
C42—C41—P2	118.9 (2)	F9—P6—F8	90.23 (11)
C43—C42—C41	120.1 (3)	F11—P6—F8	179.79 (14)
C43—C42—H42	120	F7—P6—F12	90.42 (13)
C41—C42—H42	120	F10—P6—F12	89.94 (12)
C44—C43—C42	120.0 (3)	F9—P6—F12	179.60 (14)
C44—C43—H43	120	F11—P6—F12	89.97 (11)
C42—C43—H43	120	F8—P6—F12	89.87 (11)
C43—C44—C45	120.1 (3)	P2—Pt1—P3	178.92 (3)
C43—C44—H44	120	P2—Pt1—P4	108.78 (3)
C45—C44—H44	120	P3—Pt1—P4	70.64 (3)
C44—C45—C46	120.6 (3)	P2—Pt1—P1	70.45 (3)
C44—C45—H45	119.7	P3—Pt1—P1	110.08 (3)
C46—C45—H45	119.7	P4—Pt1—P1	177.37 (3)
C45—C46—C41	119.7 (3)	Cl4A—C02A—Cl3	120.6 (2)
C45—C46—H46	120.2	Cl4A—C02A—H02A	107.2
C41—C46—H46	120.2	Cl3—C02A—H02A	107.2
C56—C51—C52	120.3 (3)	Cl4A—C02A—H02B	107.2
C56—C51—P3	119.5 (2)	Cl3—C02A—H02B	107.2
C52—C51—P3	120.2 (2)	H02A—C02A—H02B	106.8
			
N1—C1—C2—C3	169.4 (3)	C16—C11—P1—Pt1	−105.0 (2)
C1—C2—C3—C4	60.4 (4)	C26—C21—P1—N1	100.9 (2)
C2—C3—C4—C5	−177.3 (3)	C22—C21—P1—N1	−72.6 (3)
N2—C6—C7—C8	−177.1 (3)	C26—C21—P1—C11	−140.5 (2)
C6—C7—C8—C9	−62.8 (4)	C22—C21—P1—C11	46.0 (3)
C7—C8—C9—C10	−174.0 (3)	C26—C21—P1—Pt1	−4.0 (3)
C16—C11—C12—C13	0.5 (5)	C22—C21—P1—Pt1	−177.5 (2)
P1—C11—C12—C13	−169.1 (2)	C1—N1—P2—C41	51.9 (3)
C11—C12—C13—C14	0.3 (5)	P1—N1—P2—C41	−125.63 (15)
C12—C13—C14—C15	−0.7 (5)	C1—N1—P2—C31	−66.8 (3)
C13—C14—C15—C16	0.3 (5)	P1—N1—P2—C31	115.69 (15)
C14—C15—C16—C11	0.5 (5)	C1—N1—P2—Pt1	174.1 (3)
C12—C11—C16—C15	−0.9 (5)	P1—N1—P2—Pt1	−3.46 (12)
P1—C11—C16—C15	168.6 (3)	C46—C41—P2—N1	−79.3 (3)
C26—C21—C22—C23	0.2 (4)	C42—C41—P2—N1	103.1 (3)
P1—C21—C22—C23	173.6 (2)	C46—C41—P2—C31	42.2 (3)
C21—C22—C23—C24	0.1 (5)	C42—C41—P2—C31	−135.4 (3)
C22—C23—C24—C25	−0.2 (5)	C46—C41—P2—Pt1	175.4 (2)
C23—C24—C25—C26	0.1 (5)	C42—C41—P2—Pt1	−2.2 (3)
C24—C25—C26—C21	0.2 (5)	C32—C31—P2—N1	−40.8 (3)
C22—C21—C26—C25	−0.3 (5)	C36—C31—P2—N1	150.1 (2)
P1—C21—C26—C25	−173.8 (2)	C32—C31—P2—C41	−160.9 (3)
C36—C31—C32—C33	−2.1 (5)	C36—C31—P2—C41	30.0 (3)
P2—C31—C32—C33	−171.2 (2)	C32—C31—P2—Pt1	64.1 (3)
C31—C32—C33—C34	0.9 (5)	C36—C31—P2—Pt1	−105.0 (2)
C32—C33—C34—C35	0.2 (5)	C6—N2—P3—C61	−49.9 (3)
C33—C34—C35—C36	0.0 (5)	P4—N2—P3—C61	115.82 (14)
C34—C35—C36—C31	−1.2 (5)	C6—N2—P3—C51	67.7 (3)
C32—C31—C36—C35	2.2 (5)	P4—N2—P3—C51	−126.55 (15)
P2—C31—C36—C35	171.4 (2)	C6—N2—P3—Pt1	−165.6 (2)
C46—C41—C42—C43	−1.2 (5)	P4—N2—P3—Pt1	0.08 (12)
P2—C41—C42—C43	176.5 (3)	C66—C61—P3—N2	−36.4 (3)
C41—C42—C43—C44	−0.7 (5)	C62—C61—P3—N2	156.9 (2)
C42—C43—C44—C45	2.8 (5)	C66—C61—P3—C51	−157.2 (2)
C43—C44—C45—C46	−3.0 (5)	C62—C61—P3—C51	36.1 (3)
C44—C45—C46—C41	1.1 (5)	C66—C61—P3—Pt1	66.0 (2)
C42—C41—C46—C45	1.0 (5)	C62—C61—P3—Pt1	−100.8 (2)
P2—C41—C46—C45	−176.6 (3)	C56—C51—P3—N2	108.6 (3)
C56—C51—C52—C53	−0.7 (5)	C52—C51—P3—N2	−72.3 (3)
P3—C51—C52—C53	−179.9 (3)	C56—C51—P3—C61	−131.9 (3)
C51—C52—C53—C54	2.4 (5)	C52—C51—P3—C61	47.3 (3)
C52—C53—C54—C55	−2.4 (5)	C56—C51—P3—Pt1	0.0 (3)
C53—C54—C55—C56	0.8 (5)	C52—C51—P3—Pt1	179.1 (2)
C52—C51—C56—C55	−0.9 (5)	C6—N2—P4—C81	43.9 (3)
P3—C51—C56—C55	178.3 (2)	P3—N2—P4—C81	−121.62 (15)
C54—C55—C56—C51	0.8 (5)	C6—N2—P4—C71	−72.4 (3)
C66—C61—C62—C63	−0.2 (4)	P3—N2—P4—C71	122.07 (15)
P3—C61—C62—C63	166.6 (2)	C6—N2—P4—Pt1	165.5 (2)
C61—C62—C63—C64	0.5 (5)	P3—N2—P4—Pt1	−0.08 (12)
C62—C63—C64—C65	0.2 (5)	C82—C81—P4—N2	95.9 (3)
C63—C64—C65—C66	−1.2 (5)	C86—C81—P4—N2	−83.0 (3)
C64—C65—C66—C61	1.5 (4)	C82—C81—P4—C71	−144.1 (2)
C62—C61—C66—C65	−0.8 (4)	C86—C81—P4—C71	37.0 (3)
P3—C61—C66—C65	−167.6 (2)	C82—C81—P4—Pt1	−8.9 (3)
C76—C71—C72—C73	1.4 (5)	C86—C81—P4—Pt1	172.2 (2)
P4—C71—C72—C73	173.8 (2)	C76—C71—P4—N2	−26.7 (3)
C71—C72—C73—C74	2.1 (5)	C72—C71—P4—N2	161.2 (2)
C72—C73—C74—C75	−3.6 (5)	C76—C71—P4—C81	−145.4 (3)
C73—C74—C75—C76	1.5 (5)	C72—C71—P4—C81	42.5 (3)
C74—C75—C76—C71	2.1 (5)	C76—C71—P4—Pt1	79.5 (3)
C72—C71—C76—C75	−3.5 (5)	C72—C71—P4—Pt1	−92.7 (2)
P4—C71—C76—C75	−175.5 (3)	N1—P2—Pt1—P4	179.95 (9)
C86—C81—C82—C83	−0.3 (4)	C41—P2—Pt1—P4	−65.57 (12)
P4—C81—C82—C83	−179.2 (2)	C31—P2—Pt1—P4	63.88 (13)
C81—C82—C83—C84	−0.4 (5)	N1—P2—Pt1—P1	2.62 (9)
C82—C83—C84—C85	1.3 (5)	C41—P2—Pt1—P1	117.10 (12)
C83—C84—C85—C86	−1.5 (5)	C31—P2—Pt1—P1	−113.45 (13)
C84—C85—C86—C81	0.7 (5)	N2—P3—Pt1—P4	−0.06 (9)
C82—C81—C86—C85	0.2 (4)	C61—P3—Pt1—P4	−112.91 (11)
P4—C81—C86—C85	179.1 (2)	C51—P3—Pt1—P4	117.99 (14)
C2—C1—N1—P2	81.3 (4)	N2—P3—Pt1—P1	177.25 (9)
C2—C1—N1—P1	−101.7 (3)	C61—P3—Pt1—P1	64.40 (11)
C7—C6—N2—P4	102.6 (3)	C51—P3—Pt1—P1	−64.70 (14)
C7—C6—N2—P3	−95.2 (3)	N2—P4—Pt1—P2	−178.97 (9)
C1—N1—P1—C11	−55.3 (3)	C81—P4—Pt1—P2	−64.89 (12)
P2—N1—P1—C11	122.28 (14)	C71—P4—Pt1—P2	64.58 (12)
C1—N1—P1—C21	62.7 (3)	N2—P4—Pt1—P3	0.06 (9)
P2—N1—P1—C21	−119.69 (15)	C81—P4—Pt1—P3	114.14 (11)
C1—N1—P1—Pt1	−174.2 (3)	C71—P4—Pt1—P3	−116.39 (12)
P2—N1—P1—Pt1	3.44 (12)	N1—P1—Pt1—P2	−2.62 (9)
C12—C11—P1—N1	−38.5 (3)	C11—P1—Pt1—P2	−115.62 (12)
C16—C11—P1—N1	151.8 (2)	C21—P1—Pt1—P2	110.97 (11)
C12—C11—P1—C21	−156.8 (2)	N1—P1—Pt1—P3	178.39 (9)
C16—C11—P1—C21	33.5 (3)	C11—P1—Pt1—P3	65.38 (12)
C12—C11—P1—Pt1	64.7 (3)	C21—P1—Pt1—P3	−68.03 (12)

### Hydrogen-bond geometry (Å, °)

**Table:**

*D*—H···*A*	*D*—H	H···*A*	*D*···*A*	*D*—H···*A*
C25—H25···F8	0.95	2.53	3.437 (4)	160
C55—H55···F2	0.95	2.49	3.079 (4)	120
C65—H65···F10	0.95	2.47	3.297 (4)	145
C83—H83···F11	0.95	2.37	3.267 (4)	158
C01—H01B···F2^i^	0.99	2.29	3.244 (5)	161
C01—H01B···F6^i^	0.99	2.4	3.150 (5)	132
C5—H5C···F11^ii^	0.98	2.51	3.196 (4)	127
C8—H8A···F3^iii^	0.99	2.54	3.450 (4)	153
C53—H53···F7^iv^	0.95	2.51	3.273 (4)	137
C63—H63···F4^v^	0.95	2.51	3.373 (4)	151
C73—H73···F9^vi^	0.95	2.53	3.426 (4)	156

Symmetry codes: (i) *x*+1/2, −*y*+3/2, *z*−1/2; (ii) *x*−1, *y*, *z*; (iii) *x*+1, *y*, *z*; (iv) *x*, −*y*+1, *z*+1/2; (v) *x*+1/2, *y*−1/2, *z*; (vi) *x*−1/2, *y*+1/2, *z*.
